# Pain perception during debridement of hypersensitive teeth elicited by two ultrasonic scalers

**DOI:** 10.1007/s00784-016-1971-4

**Published:** 2016-10-15

**Authors:** S. Müller, H. Huber, G. Goebel, G. Wimmer, I. Kapferer-Seebacher

**Affiliations:** 1 Department of Dental Prosthetics and Restorative Dentistry, Medical University of Innsbruck, Anichstr. 35, 6020 Innsbruck, Austria; 2 Department of Medical Statistics, Informatics and Health Economics, Medical University of Innsbruck, Schöpfstr. 41, 6020 Innsbruck, Austria; 3 Department of Restaurative Dentistry, Periodontology and Prosthodontics, Medical University of Graz, Billrothgasse 4, 8010 Graz, Austria

**Keywords:** Debridement, Pain, Ultrasonic scaler, Supportive periodontal therapy, Non-surgical periodontal therapy, Visual analogue scale

## Abstract

**Objectives:**

The ultrasonic NO PAIN technology (Electro Medical Systems, Nyon, CH) promises minimal pain during debridement due to linear oscillating action combined with a sinusoidal power output and feedback control. The aim of the present study was to measure pain perception on a visual analogue scale (VAS) during supportive periodontal therapy including debridement of hypersensitive teeth. Two ultrasonic scalers were used, one with and one without NO PAIN technology.

**Material and methods:**

In a randomized-controlled clinical study with split-mouth design, 100 hypersensitive teeth matched for air blast hypersensitivity were either treated with the ultrasonic device Piezon Master 700 or the Mini Piezon (both EMS, Nyon, CH). Pain perception during debridement was assessed by a VAS (range 0–10).

**Results:**

The average VAS for the test device Piezon Master 700 with NO PAIN technology was 3.16 ± 2.10, and for the control device Mini Piezon without NO PAIN technology 3.40 ± 2.59 (*p* = 0.490). Placing an arbitrary threshold at the VAS score of 3 for significant pain experience, 60 % of the subjects experienced no significant pain with either instrument.

**Conclusion:**

No statistically significant difference in perceived pain between the instruments used was found.

**Clinical relevance:**

Both ultrasonic devices showed very small pain intensities during debridement of highly hypersensitive teeth and can therefore be recommended for supportive periodontal therapy.

## Background

The Consensus report of the 11th European Workshop on Periodontology on effective prevention of periodontal and peri-implant diseases reinforced the need to enrol patients treated for periodontitis in a supportive periodontal therapy regimen [1]. Supportive periodontal therapy aims at preventing the recurrence of periodontal disease in terms of tooth loss and additional attachment loss through periodic preventive interventions [2, 3]. Such regimen includes routine assessments of disease and oral hygiene status, behaviour modification and professional mechanical plaque and calculus removal (PMPR) [2]. The importance and effectiveness of supportive periodontal therapy in the secondary prevention of periodontal disease have been well established [1]. The authors of the Consensus report of the 11th European Workshop on Periodontology concluded that patients treated for periodontitis can maintain their dentition with limited variations in periodontal parameters when regularly complying with a supportive periodontal therapy regimen based on routine PMPR [1]. Additionally, patients irregularly complying with the planned supportive periodontal therapy regimen showed greater tooth loss and disease progression when compared to patients who comply regularly [1, 4].

Pain during PMPR was recently reported to be a significant factor influencing clinical compliance to periodontal therapy [5]. The healing of periodontal tissues after active periodontal therapy often results in gingival recession, and in addition, root debridement leads to loss of cementum [6, 7]. The short, sharp pain arising from exposed dentin in response to thermal, tactile, osmotic or chemical stimuli has been defined as dentin hypersensitivity [8]. Levels of dentin hypersensitivity may increase after surgical as well as non-surgical periodontal treatment [9–11]. Among periodontal patients, the occurrence of root sensitivity has been reported to reach up to 98 % [12]. PMPR often induces dentin hypersensitivity due to thermal or tactile stimuli. The ability to deliver dental care with a minimum of patient discomfort should be an essential part of a clinician’s skills to avoid a decline of compliance with supportive periodontal therapy [13].

The EMS Piezon® NO PAIN technology (Electro Medical Systems (EMS), Nyon, CH) promises minimal pain during PMPR and no injury of the gingiva, due to controlled linear oscillating instrument movements parallel to the tooth surface, combined with a sinusoidal power output and feedback control [14].

The aim of the present randomized-controlled clinical study was to compare subjective pain intensities during PMPR of hypersensitive teeth with two piezoelectric ultrasonic devices, one including NO PAIN technology (Piezon Master 700, EMS, Nyon, CH), and one without (Mini Piezon, EMS, Nyon, CH).

## Material and methods

### Ethical considerations

The Ethics Committee of the Medical University of Innsbruck, Austria, approved the study. The study was conducted in accordance with the 1964 Helsinki declaration and its later amendments. All subjects signed an informed written consent prior to the study enrolment.

### Study subjects

**Fig. 1:**
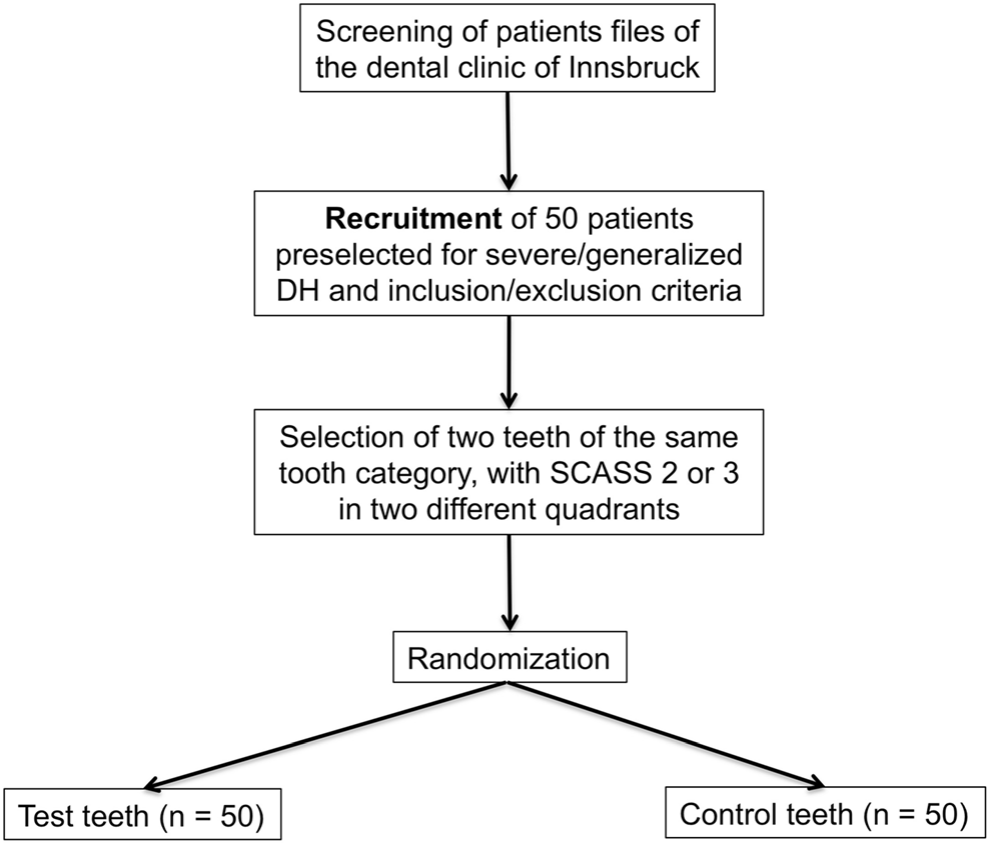
Patient flow chart. Patients were preselected by their dentist/student regarding inclusion and exclusion criteria. Only patients known for generalized and severe dentin hypersensitivity were recruited for the clinical trial

For the study, 53 patients of the dental clinic of the Medical University of Innsbruck who were known for generalized and severe dentin hypersensitivity were recruited (Fig. 1). Subjects had to exhibit a minimum of two hypersensitive teeth in two different quadrants. For the respective teeth, an air blast stimuli score of 2 or 3 (Schiff Cold Air Sensitivity Scale - SCASS) had to be present at the baseline examination. Both teeth of the subject had to feature the same air blast stimuli score. Additionally, test and control teeth had to be from the same tooth category (category 1: incisive, category 2: canines and premolars, category 3: molars). Any teeth with cracked enamel, enamel defects, caries, or extensive/defective restorations, clinically diagnosed pulpitis, and teeth with orthodontic appliances were excluded. Additional exclusion criteria encompassed subjects with gross oral pathology, PMPR or orthodontic treatment within the last 3 months, subjects with eating disorders, as well as pregnant or lactating women, and psychiatric disorders. Current users of anticonvulsants, antihistamines, antidepressants, sedatives, tranquillisers, anti-inflammatory drugs or daily analgesics were also excluded.

### Clinical intervention

At baseline, one investigator (HH) measured tactile and air blast hypersensitivity and selected two hypersensitive teeth in two different quadrants with SCASS 2 or 3. For SCASS, the test and control teeth were isolated from the adjacent teeth by the placement of red boxing wax. Air was delivered from a standard dental unit air syringe at maximal pressure (45 psi) and at an environmental temperature of 19–24°. Air was applied for 1 s at a distance of 1 cm perpendicular to the buccal surface of the tooth. The SCASS was used to assess the subject’s response to the stimulus. The scale is graduated into four units: 0 = subject does not respond to the stimulus; 1 = subject does not respond to the stimulus, but considers stimulus to be painful; 2 = subject responds to air stimulus, but does not move away from the stimulus; 3 = subject responds significantly to air stimulus, moves away from the stimulus, and requests immediate termination of the stimulus [15]. Patients were informed before testing about the different units of the scale. Tactile hypersensitivity was assessed by scratching on the dentinal surface with a sharp-tipped probe and a maximum pressure of 70 g. Pressure of 70 g was calibrated with a letter balance before each investigation. The subjects graded pain intensity on a visual analogue scale (VAS) (0 = no pain and 10 = extreme, unbearable pain). Patients were instructed to point at the VAS. Probing pocket depths recorded at six sites per tooth were available from all patients. Recession depth was measured on the buccal aspect of the tooth.

Both teeth were matched for air blast hypersensitivity and were randomly assigned by the second investigator (MS) to one of two treatment groups by rolling a dice: (1) supragingival debridement for 30 s using an ultrasonic scaler with the specific NO PAIN technology (Piezon Master 700, EMS, Nyon, CH), or (2) supragingival debridement for 30 s using an ultrasonic scaler without the NO PAIN technology (Mini Piezon, EMS, Nyon, CH). For both devices, the same tip was used (EMS Instrument PS). According to oral advice of the manufacturer, the power of both devices was set to 50 %. Temperature of physiological saline solution and water for cooling was adjusted to 24 °C. During debridement, the lower end of the tip was applied from coronal to apical with minimal pressure using brushing strokes parallel to the tooth surface. The blinded investigator HH performed the follow-up examinations: Intervention blinded patients were asked to protocol their perception of the instrumentation immediately after the treatment (main outcome measure) on an interval scale (VAS) ranging from 0, representing no pain or discomfort, to 10, representing maximum pain and discomfort.

### Statistical analyses

Standard descriptive methods were used to summarize the parameters studied. The Wilcoxon signed-rank test was used to evaluate differences between values for pain perception during PMPR (VAS, main outcome). Differences of baseline hypersensitivity levels between treatment groups were evaluated with the chi-square test (SCASS) or Wilcoxon signed-rank test (tactile hypersensitivity, VAS). All statistical tests of the hypotheses were two-sided, and a level of significance of alpha = 0.05 was employed.

## Results

### Baseline data

One hundred teeth in 50 subjects (31 females, 19 males) were enrolled in the study. All participants were Caucasians, aged 20–79 years (mean age ± SD = 44.84 ± 14.06). Fifty-six percent of the subjects had never smoked before, and 44 % were smokers. Thirty-six subjects showed baseline SCASS 2 on matched test and control teeth, and 14 subjects exhibited with SCASS 3 on matched test and control teeth. Six subjects with tooth category 1, 27 subjects with tooth category 2, and 17 subjects with tooth category 3 were included. There was no statistical significant difference between test and control teeth in baseline tactile hypersensitivity (VAS baseline 1.60 ± 2.09 and 1.62 ± 2.19, respectively; *p* = 0.617) (Table 1).

**Table 1:** Dentin hypersensitivity at baseline and pain perception during debridement of test and control teeth

	Piezon Master 700 (*n* = 50)	Mini Piezon (*n* = 50)
Tooth categories		
Category 1: incisives, *n*	6	6
Category 2: canines and premolars, *n*	27	27
Category 3: molars, *n*	17	17
Periodontal parameters		
Probing pocket depth, *m ± sd*	3.43 ± 1.44	3.40 ± 1.47
Recession depth, *m ± sd*	2.03 ± 1.27	2.30 ± 1.26
Airblast sensitivity scale		
SCASS 2, *n*	36	36
SCASS 3, *n*	14	14
Tactile sensitivity scale, baseline		
Visual analogue scale, *m ± sd*	1.60 ± 2.09	1.62 ± 2.19
Pain perception during debridement		
Visual analogue scale, *m ± sd*	3.16 ± 2.10	3.40 ± 2.59

One hundred teeth in 50 subjects (31 females, 19 males; mean age ± SD = 44.84 ± 14.06) were enrolled in the study. Fifty-six percent of the subjects had never smoked before, and 44 % were smokers. Two teeth in two different quadrants were matched for SCASS and tooth category

*n* number, *SCASS* Schiff Cold Air Sensitivity Scale, *m* mean, *sd* standard deviation

**Fig. 2:**
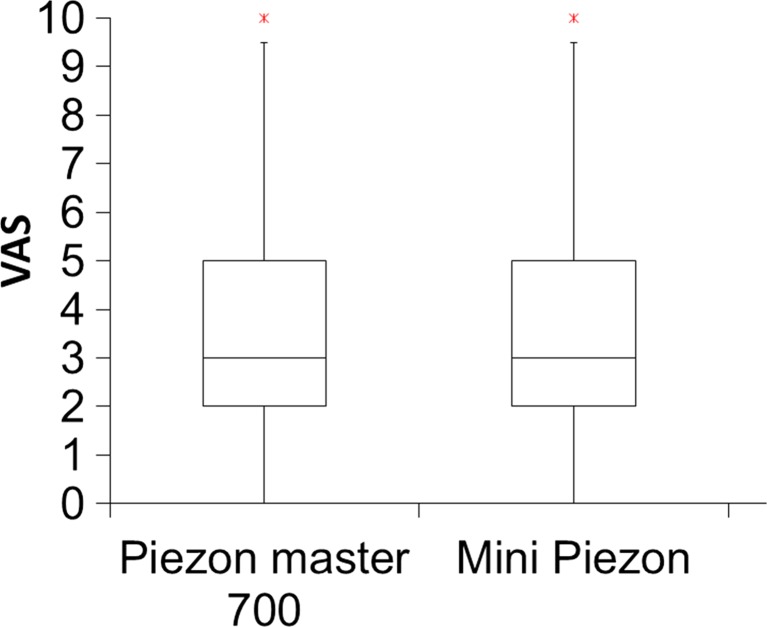
Box plot for the pain perception during debridement (median, outliers, 10, 25, 75, and 90 % percentiles). Pain perception of the instrumentation was assessed immediately after the treatment on an interval scale (visual analogue scale, *VAS*) ranging from 0, representing no pain or discomfort, to 10, representing maximum pain and discomfort. With a median of 3, pain perception was low for both devices

No statistically significant difference in perceived pain between the instruments used was found. For the test device Piezon Master 700 with NO PAIN technology, the average VAS value during debridement was 3.16 ± 2.10, and for the control device Mini Piezon without NO PAIN technology, this was 3.40 ± 2.59 (*p* = 0.490). The median was 3 for both instruments (range 1–10) (Fig. 1). Placing an arbitrary threshold for significant pain experience at the VAS score of 3 [16], 60 % (*n* = 30) of the subjects experienced no significant pain (VAS 0 to 3) with either instrument. Further assuming another arbitrary limit at 7 [16], two subjects perceived great pain (VAS 7 to 10) during treatment with the Piezon Master 700, and seven patients perceived great pain during treatment with the Mini Piezon (Fig. 2).

## Discussion

Many studies have highlighted the importance of regular supportive periodontal therapy including PMPR. Among the most well-known studies are Hirschfield and Wasserman [17], McFall [18], Lindhe and Nyman [19], Goldman et al. [20], and Axelsson and Lindhe [21]. Indeed, there is a significant increase in tooth loss in non-compliers or irregular compliers compared to compliers [22]. Incidence of new sites with probing depth of > or =5 mm varied between 3.2 % for the compliant and 5.8 % for the non-compliant patients (mean delay from the scheduled recall sessions: compliant within 1–6 weeks; and not compliant >6 weeks) [23]. A painless treatment increases patient comfort during PMPR, and might therefore increase patient compliance [5]. This in turn may provide a better long-term prognosis for periodontal therapy.

In the present randomized-controlled and double blind trial, 100 teeth with dentin hypersensitivity were enrolled. Two teeth in each subject were matched according to air blast hypersensitivity and tooth category and were randomly assigned to debridement with an ultrasonic device with or without NO PAIN technology. Debridement of hypersensitive dentin was restricted on supragingival areas to avoid pain by gingival injury, which would have falsified the result. Pain perception of the instrumentation was recorded instantly with a VAS. Verbal reports are known to be shaped by a variety of psychosocial variables. Additionally, pain is not a simple sensory state but is influenced by cultural learning, the meaning of the situation, attention and other psychological variables [24]. Therefore, to overcome inter-individual differences between test and control patients, we investigated both devices in a split-mouth clinical trial. To overcome intra-individual differences in pain perception between different teeth, tooth categories and air blast hypersensitivity were matched between test and control teeth.

**Fig. 3:**
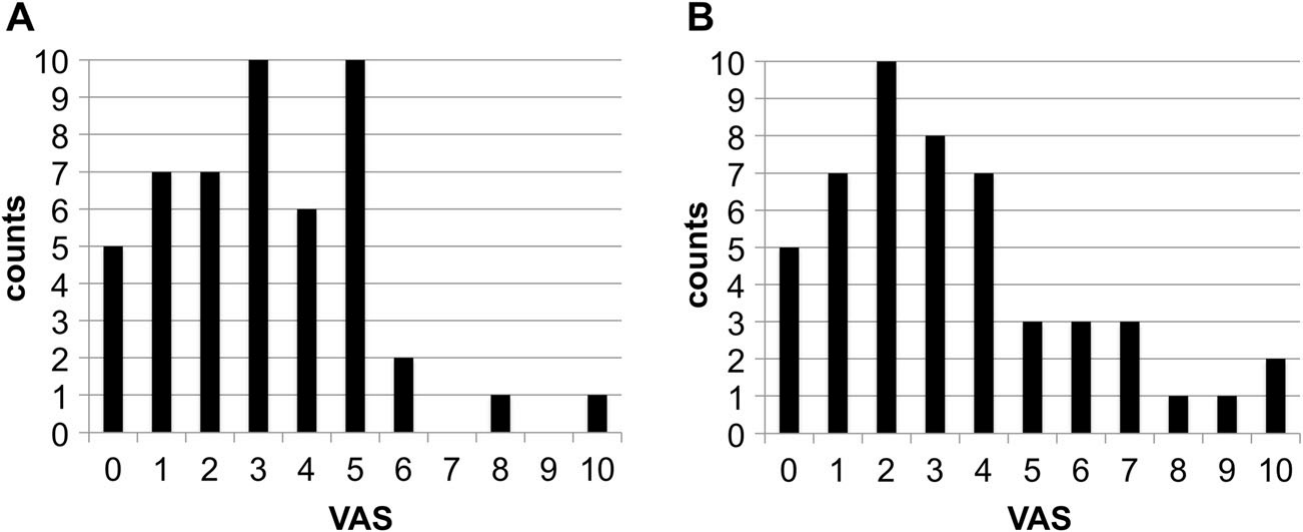
Frequency distribution of VAS scores for the treatment with **a** the Piezon Master 700 and **b** the Mini Piezon (both EMS, Nyon, CH). Pain perception of the instrumentation was assessed immediately after the treatment on an interval scale (visual analogue scale, *VAS*) ranging from 0, representing no pain or discomfort, to 10, representing maximum pain and discomfort. Placing an arbitrary threshold at the *VAS* score of 3, 60 % (*n* = 30) of the subjects experienced no significant pain with either instrument. Further assuming another arbitrary limit at 7, two subjects perceived great pain during treatment with the Piezon Master 700, and seven patients perceived great pain during treatment with the Mini Piezon

Pain perception during instrumentation was low for both ultrasonic devices (VAS median 3), and there was no statistically significant difference between the two treatment modalities. More subjects (*n* = 7) perceived great pain (VAS 7–10) with the Mini Piezon compared to subjects exposed to the Piezon Master 700 (*n* = 2) (Fisher Exact Probability Test: *p* = 0.159). Our results are in line with previous studies on pain perception during debridement with piezoceramic ultrasonic devices. Braun et al. compared pain intensities during debridement with hand instruments (Gracey-curettes, Hu-Friedy, Leimen, Germany), a piezo ultrasonic instrument (SirosonTMS, instrument N°3, Siemens, Bensheim, Germany) or the Vector™-system (Duerr Dental, Bietigheim-Bissingen, Germany); mean VAS values for pain perception during therapy was 3.7 ± 1.8 for the piezo ultrasonic device [25]. Kocher et al. compared pain intensities during debridement with a sonic (Sonicflex2000, KaVo, Biberach, Germany) and a piezoceramic ultrasonic scaler (PiezonMaster 400, EMS, Nyon, CH) in a split-mouth design; the median VAS was 3 for both instruments [16]. Kocher et al. concluded that the motion of the instrument’s tip might be redundant with respect to perceived pain [16]. The EMS Piezon® NO PAIN technology (Electro Medical Systems (EMS), Nyon, CH) promises minimal pain during PMPR and no injury of the gingiva, due to controlled linear oscillating instrument movements parallel to the tooth surface, combined with a sinusoidal power output and feedback control [14]. Emmelmann studied in his thesis the motion of instrument tips in an unloaded and loaded mode with a high speed camera with 7500 frames-per-second and provided first evidence that the instrument’s tip (EMS® Instrument P) of the Piezon Master 700 has a slight elliptic motion [26]. Possibly, the motion of the instrument’s tip with the Piezon Master 700 is very similar to the Mini Piezon, which is also equipped with the Piezon® technology (Fig. 3). The feedback control does not seem to have a significant effect on pain reduction in supportive periodontal therapy where little calculus is present.

In conclusion, both ultrasonic devices investigated in the present study showed very small pain intensities during debridement of highly hypersensitive teeth and can therefore be recommended for supportive periodontal therapy.
